# Open Space Loss and Land Inequality in United States' Cities, 1990–2000

**DOI:** 10.1371/journal.pone.0009509

**Published:** 2010-03-03

**Authors:** Robert I. McDonald, Richard T. T. Forman, Peter Kareiva

**Affiliations:** 1 Worldwide Office, The Nature Conservancy, Arlington, Virginia, United States of America; 2 Graduate School of Design, Harvard University, Cambridge, Massachusetts, United States of America; 3 Seattle Office, The Nature Conservancy, Seattle, Washington, United States of America; University of Bristol, United Kingdom

## Abstract

Urban growth reduces open space in and around cities, impacting biodiversity and ecosystem services. Using land-cover and population data, we examined land consumption and open space loss between 1990 and 2000 for all 274 metropolitan areas in the contiguous United States. Nationally, 1.4 million ha of open space was lost, and the amount lost in a given city was correlated with population growth (r(272) = 0.85, P<0.001). In 2000, cities varied in per capita land consumption by an order of magnitude, from 459 m^2^/person in New York to 5393 m^2^/person in Grand Forks, ND. The per capita land consumption (m^2^/person) of most cities decreased on average over the decade from 1,564 to 1,454 m^ 2^/person, but there was substantial regional variation and some cities even increased. Cities with greater conservation funding or more reform-minded zoning tended to decrease in per capita land consumption more than other cities. The majority of developed area in cities is in low-density neighborhoods housing a small proportion of urban residents, with Gini coefficients that quantify this developed land inequality averaging 0.63. Our results suggest conservation funding and reform-minded zoning decrease per capita open space loss.

## Introduction

Urban population in the United States has climbed dramatically in recent decades, from 84.5 million in 1950 to 226 million in 2000 [1]. At the same time, many of these new residents settled in suburbs farther from the city center at relatively low density [2], resulting in a drastic expansion in urbanized area [3], [4]. The expansion of urban area can have significant ecological impacts [5]–[7]. The amount of open space is reduced [8], fragmenting natural habitat as well as reducing the recreational and other amenities people can enjoy from open space [9]–[11]. Open space is defined here as agricultural land and more natural land-cover such as forest and grassland, including both remnant patches within a city as well as larger patches at the city's fringe.

Rapid urban growth has raised public concern about the ecological and social impacts of “sprawl,” which has been defined as new settlements relatively far from city centers, as opposed to “densification,” which raises the density of already developed neighborhoods [2]. One manifestation of this concern is the rapid growth of ballot initiatives to authorize bonds to fund the protection of open space. Another manifestation is the attempt to limit sprawl through reform-minded zoning and other parts of the “smart growth” [12], [13] or “New Urbanist” [14]–[16] agenda. Reform-minded zoning is characterized by efforts to restrict development and sometimes to channel it to existing urban areas and can take many forms, such as urban growth boundaries like in Portland, Oregon, or changed zoning codes that encourage multi-use construction and discourage car travel.

There have been dozens of books and hundreds of papers about sprawl [2], [3], [17]–[22]. However, quantitative and comprehensive assessments of sprawl are scarce. In this study we quantify the total amount of open space lost to development for US cities, as well as the developed area per capita, which we refer to as “per capita land consumption.” Moreover, most studies that have compared the density of development in different cities have used city-wide average, rather than tracking the full spectrum of development types within an urban area. Of particular interest is to what extent a small fraction of households consume large amounts of developed area while the rest of the population is concentrated in dense settlements that use much less land per capita. We quantify land inequality using the Gini index (G), which varies from 0 (most equal) to 1 (most unequal). Measurement of this “land inequality” quantifies the heterogeneity of complex urban landscapes. Note that per capita land consumption and land inequality are independent quantities, and it is possible for a city with high per capita land consumption to have either low or high levels of land inequality.

**Figure 1:**
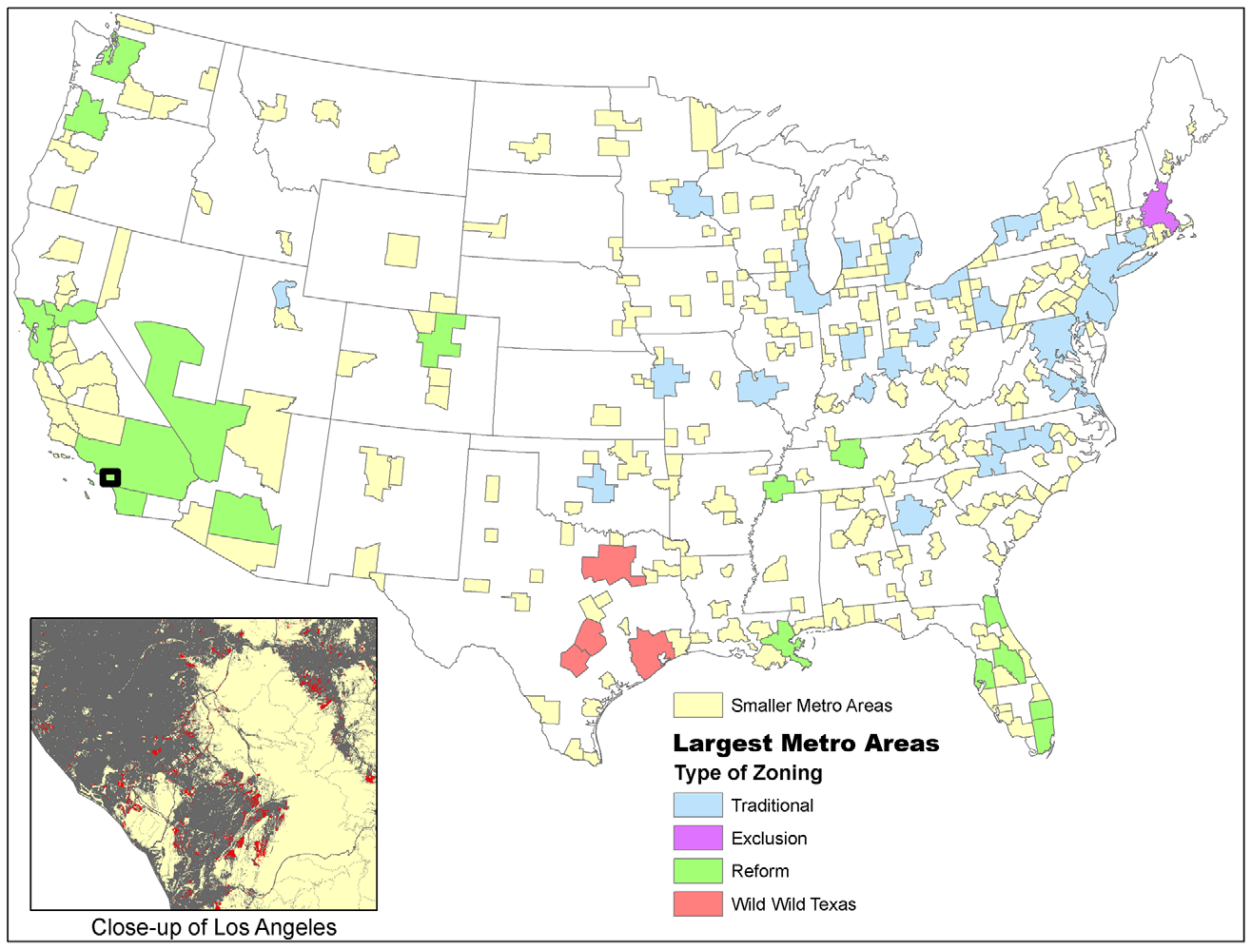
Metropolitan areas examined in this study. For the largest metropolitan areas, the type of zoning scheme is shown (see text for details). Inset shows land-cover data for the Los Angeles area, with areas built up in 1990 (gray), areas of new development between 1990 and 2000 (red), and open space (beige). For reference, the extent of the inset map is shown on the national map with a black box.

In this paper we measure the open space lost from urban growth in all 274 metropolitan statistical areas (MSAs) in the contiguous United States from 1990 to 2000 (Figure 1). For brevity, we will sometimes refer to these MSAs as “cities,” although we recognize that often one metropolitan statistical area contains multiple municipalities. We use US Census Bureau data to calculate explicit estimates of land inequality using measures commonly employed by economists studying income inequality. Finally, we look at whether the type of zoning regulations (classified as either “Traditional”, “Exclusion”, “Reform”, or “Wild Wild Texas” zoning following [23]) or the level of conservation funding affects open space loss or land inequality. We present our results in two steps, first discussing MSA-wide metrics that summarize some aspect of pattern into a single number for each MSA and then discussing fine-scale metrics that quantify variation in pattern within each MSA.

## Results

### MSA-Wide Analysis

**Figure 2:**
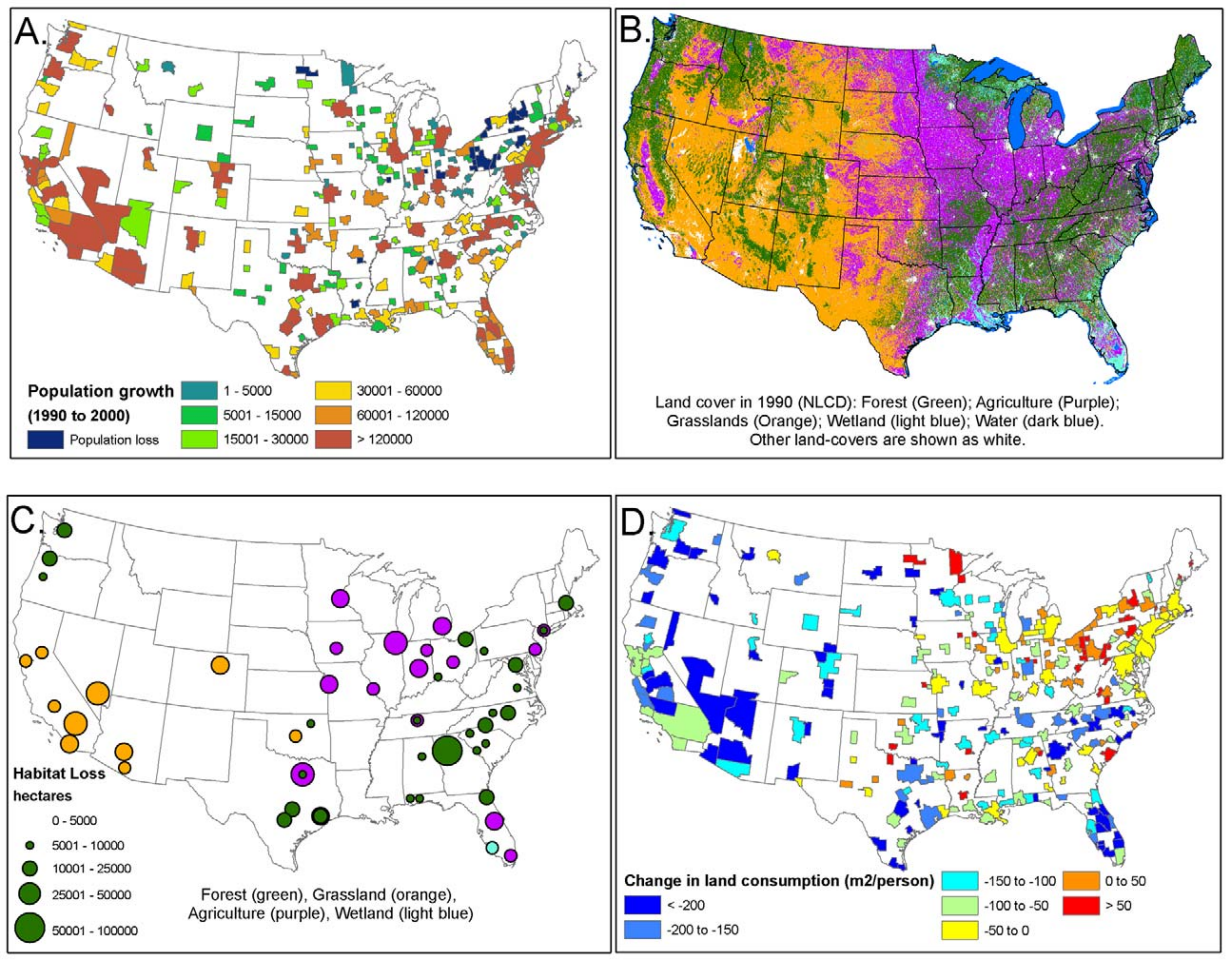
Change in population and land-cover, 1990–2000. A.) Population growth for each metropolitan area (MSA). B.) Land-cover in 1990. C.) Open space loss within each MSA. The size of the bubble indicates the amount of open space lost, and the color indicates the dominant type of land-cover lost. Cities are only marked when greater than 5000 ha was lost in at least one land-cover category. D.) The change in per-capita land consumption from 1990 to 2000. Cities with negative numbers used less land per-capita in 2000 than in 1990, while cities with positive numbers used more land per-capita.

Most MSAs in the United States increased in population from 1990 to 2000 (Figure 2A), with the greatest increase in southern California, the New York MSA, and the Atlanta MSA. Cities in the “Rust Belt,” running from upstate New York through western Pennsylvania and to Ohio, lost population. Land-cover in 1990 varied greatly across the country (Figure 2B), with the Midwest dominated by agriculture and the West dominated by grassland/shrublands. In contrast, the eastern U.S. and the Pacific Northwest are dominated by forests, while wetlands occur at greatest extent in Florida and the Gulf coast. The type of open space lost to urban development (Figure 2C) generally reflects the dominant habitat in the local region (Figure 2B). The amount of open space lost is strongly correlated with change in population (r(272) = 0.85, P<0.001), with cities with a greater increase in population losing more open space.

American cities vary by more than an order of magnitude in their MSA-wide per capita land consumption. Generally large cities have small per capita land consumption, with the five smallest in 2000 being New York (459 m^2^/person), Miami (476 m^2^/person), Philadelphia (519 m^2^/person), Los Angeles (535 m^2^/person), and Washington, DC (536 m^2^/person). Conversely, many small cities have large per capita land consumption, with the five biggest in 2000 being Grand Forks, ND (5394 m^2^/person), Bismark, ND (3913 m^2^/person), Flagstaff, AZ (3381 m^2^/person), Enid, OK (3249 m^2^/person), and Cheyenne, WY (3073 m^2^/person).

Most cities decreased in per capita land consumption from 1990 to 2000, measured in developed ha per person (Figure 2D). The average decrease in per capita land consumption was 110 m^2^/person, but some cities increased and some cities decreased, from a decline of 939 m^2^/person in Naples, FL, to an increase of 316 m^2^/person in Grand Forks, ND. Out of cities with more than a million people in 2000, the city with the greatest decrease in per capita land consumption was Las Vegas (a decrease of 577 m^2^/person), while the city with the greatest increase in per capita land consumption was Pittsburgh (an increase of 47 m^2^/person).

Greater amounts of conservation funding are associated with a greater decrease in per capita land consumption (Table 1; F(3, 270) = 3.8, P = 0.01). Although per capita land consumption generally declined across most cities, those cities with no conservation funding from 1990–2000 (N = 199) showed a significantly smaller decrease than do cities with high (>$100/person; N = 20) levels of conservation funding. Cities with low ($0–$25/person; N = 25) and moderate ($25–$100/person; N = 30) levels of conservation funding vary considerably and are not significantly different from either those cities with no conservation funding or those cities with high levels of conservation funding.

**Table 1:** Conservation funding and change in per capita land consumption.

Conservation funding per capita	Change in per capita land consumption (m^2^/person)
None	−97^ a^
$0–$25	−90^a,b^
$25–$100	−151^a,b^
More than $100	−206^b^

Conservation funding is the sum of all approved municipal, special district, and county ballot initiatives from 1990–2000, in nominal $, divided by the MSA population in 1990. Changes in per capita land consumption are measured as the difference between 1990 and 2000 per capita consumption, in developed area per person. Negative numbers indicate per capita land consumption declined over the decade. Statistically different groups are shown with different superscripts.

Among the largest MSAs, the type of zoning system was correlated with the change in per capita land consumption (Table 2; F(3, 45) = 2.8, P = 0.05). The decrease in per capita land consumption for “Reform” cities (N = 17), which tend to use zoning to promote denser, mixed-use development, was significantly greater than for “Traditional” cities (N = 27), which tend to use zoning to separate competing land-uses and protect property values. The “Exclusion” (N = 1) and “Wild Wild Texas” (N = 4) categories had few MSAs, and results for these groups were not statistically different from either “Reform” or “Traditional” cities.

**Table 2:** The type of zoning and the change in per-capita land consumption.

Zoning category	Change in per-capita land consumption (m^2^/person)
Exclusion	−33^a,b^
Traditional	−69^a^
Reform	−153^b^
Wild Wild Texas	−153^a,b^

See text for the details of the four zoning categories used. Changes in per-capita land consumption are measured as the difference between 1990 and 2000 per capita land consumption, in developed area per person. Negative numbers indicate per-capita land consumption declined over the decade. Statistically different groups are shown with different superscripts.

### Fine-Scale Analysis

#### Neighborhood density

**Figure 3:**
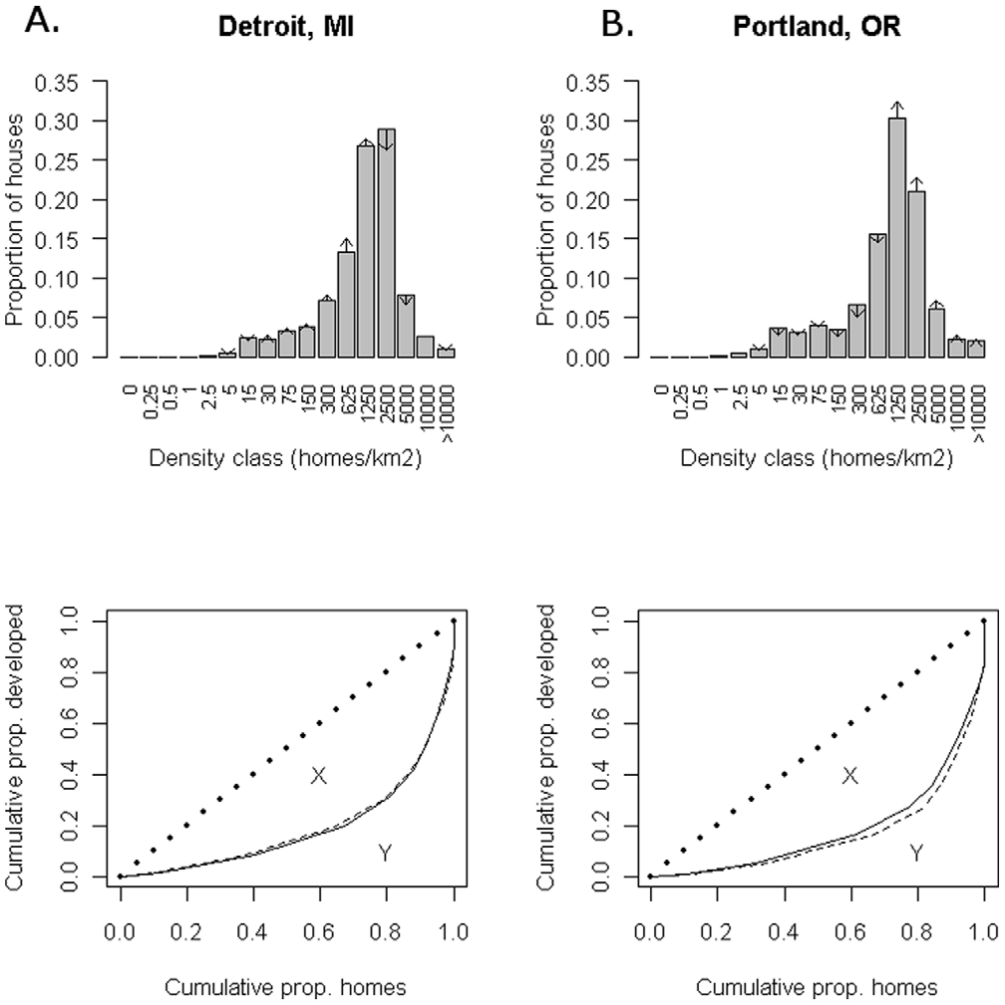
The distribution of household density, 1990–2000. A.) The proportion of households in neighborhoods of different density categories in 1990 in two cities, Detroit, MI and Portland, OR. Changes in the proportion in each category over the decade are shown by the length of the arrows. For clarity, arrows are only drawn when there is a change of at least 0.5%. For instance, in Detroit almost a third of households are in neighborhoods from 1250–2500 homes/km^2^, but this proportion is decreasing over time. B.) The cumulative proportion of development versus the cumulative proportion of homes in 1990 (solid line) and 2000 (dashed line). For example, in Detroit the 80% of homes in the highest density neighborhoods consume only 35% of total developed area. For reference, the line of perfect equality is shown (dashed). If all households were in neighborhoods of equal density, the empirical curve would fall along this line of perfect equality. The Gini coefficient is defined as area X divided by the total area under the line of perfect equality (X+Y).

**Figure 4:**
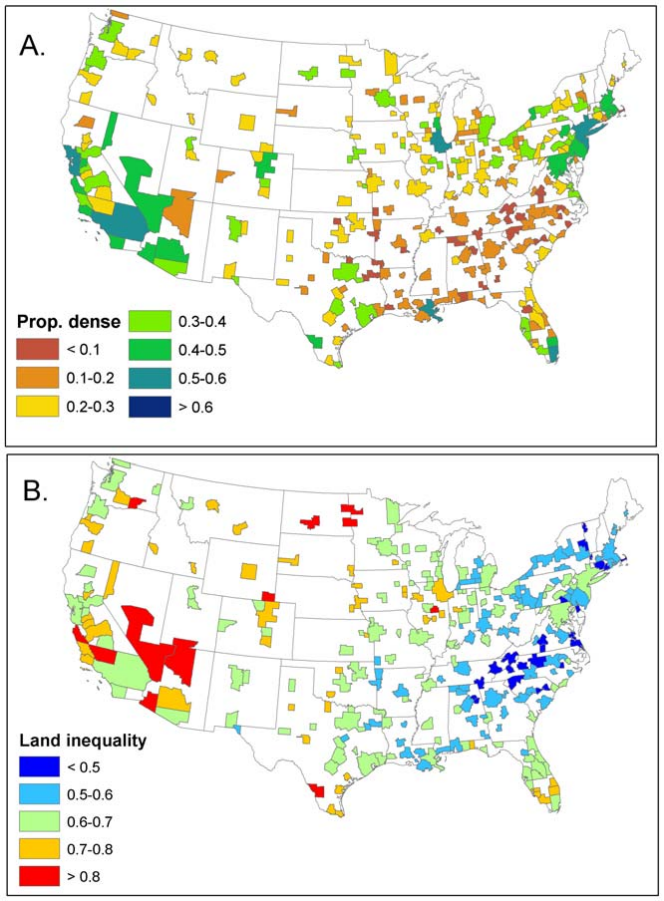
Fine-scale pattern of household density in U.S. cities in 2000. A.) The proportion of households in neighborhoods that have a density greater than 1250 houses/km^2^. B.) Land inequality in U.S. cities, as measured by the Gini coefficient, which ranges from 0–1. Higher values indicate greater land inequality.

In most American cities, the distribution of neighborhood density is similar to the patterns for Detroit, MI and Portland, OR (Figure 3A). Only a small percentage of homes are located in census blocks with very low or very high housing density, with most homes in neighborhoods at a density of 300–2500 units/km^2^. The percentage of houses in 2000 in “high-density” neighborhoods with greater than 1250 units/km^2^ (Figure 4A) is greatest in the New York MSA, at 63%. Generally the Northeast and the West Coast have fairly dense cities by this metric, while the Southeast (excluding Florida) has a low proportion of housing in dense neighborhoods. Bigger cities have a greater proportion of housing in dense neighborhoods (r(272) = 0.52, P<0.001), as do cities with a greater median house value (r(272) = 0.53, P<0.001).

The proportion of housing in dense neighborhoods is statistically related to the level of conservation funding (F(3,270) = 20.9, P<0.001). Cities with any level of conservation funding are denser than cities with no conservation funding, although the reasons for this correlation are unclear. Conservation funding may force developments to be denser by setting aside some land as off limits to development. Alternatively, or in addition, cities that care about conservation enough to approve ballot initiatives may have citizens who prefer living in denser neighborhoods. In contrast to the correlation between conservation funding and density, zoning type is not significantly correlated with the proportion of housing in dense neighborhoods.

#### Change in neighborhood density

Over the decade, there was a slight decline in the proportion of houses in Detroit that were in density classes greater than 1250 units/km^2^ while there were proportionally more houses in the smaller density categories (Figure 3A). This is a common pattern in the Midwest and East. In contrast, in West Coast cities like Portland there was a slight increase in the proportion of houses that were in larger density classes, while there were proportionally fewer houses in the smaller density classes. Cities with a greater decrease in MSA-level per capita land consumption have a greater increase in the proportion of houses in dense neighborhoods (r(272) = −0.42, P<0.001).

The zoning type was statistically related to the increase in proportion of houses in high-density neighborhoods (F(3,45) = 4.8, P = 0.006). The only significant contrast is between the “Reform” and “Traditional” zoning categories, with “Reform” cities slightly increasing in the proportion of houses in high-density neighborhoods while “Traditional” cities decreased. The change in proportion of houses in high density neighborhoods was not correlated with the median house value, the city size, or the degree of conservation funding.

#### Land inequality

Neighborhoods containing a relatively small proportion of urban residents at low density usurp most of the developed area in a city. For instance, in Detroit, MI (Figure 3B) 80% of homes are in the highest density neighborhoods that consume only about 35% of the developed area in the entire MSA. The Gini coefficient of inequality (G) is 0.63 in 2000, indicating a very unequal distribution of developed area. In Portland, OR, the distribution is slightly less unequal (G = 0.55 in 2000). Nationally, G averages 0.63 and varies from 0.35 to 0.93, with Eastern cities being more equal in their distribution of developed area than those in the Western U.S. (Figure 4B). Declining manufacturing cities in the Rust Belt and in the southern Appalachian mountains have very equal distributions, while cities in the Southwest have very unequal distributions. Cities with a higher proportion of their housing in high density neighborhoods had greater land inequality (r(272) = 0.47, P<0.001). G is not statistically related to the level of conservation funding, zoning type, the city size, or the median house value.

#### Change in land inequality

Over the decade, the Gini coefficient of land inequality did not change much. Ninety-eight cities out of the total 274 increased in G by at least 0.01, while 32 cities decreased by at least that much. Cities that increased in the proportion of houses in high-density neighborhoods had greater increases in land inequality (r(272) = 0.43, P<0.001). Cities that had a greater MSA-scale decrease in per capita land consumption had greater increases in land inequality (r(272) = −0.41,P<0.001). While absolute levels of G in 2000 were not related to conservation funding, changes in G from 1990 to 2000 were (F(3,270) = 3.8, P = 0.01). Only one contrast is marginally statistically significant, with cities with greater than $100/person spending having a greater increase in land inequality than did cities with no conservation funding. The change in G was not related to city size, the median house value, or the zoning category.

## Discussion

Our results show that patterns of urban growth from 1990 to 2000 are partially the result of contemporary factors and partially the result of historical factors. For instance, our results show that conservation funding and zoning over the decade correlated with patterns of urban growth. More conservation funding is correlated with lower per capita land consumption, an increase in the proportion of houses in dense neighborhoods, and a slight increase in land inequality. Cities with “Reform” style zoning, characterized by efforts to restrict development and sometimes to channel it to existing urban areas, also have lower per capita land consumption than other zoning styles. Note that correlation does not necessarily imply causation, and other factors might explain the development patterns in conservation friendly cities, such as their relatively fast rate of population growth or relatively high median house value. However, our results suggest that as cities protect land and tighten zoning restrictions, there is proportionally less development in more suburban areas, presumably because more dense residential development is comparatively favored by these actions. This then lowers the per-capita impact of urban residents on open space.

The process of development plays out differently in cities with different socioeconomic histories. Moreover, cultural differences exist among and within many U.S. cities, leading to varying spatial patterns of development. However, a general historical pattern exists. In many U.S. cities, an urban core existed in the decades or centuries prior to the widespread use of the automobile, and these neighborhoods have high population density and small amounts of developed area per capita. The surrounding suburban and exurban areas, created predominately after WWII, contain residents living at lower population density and consume more land per capita [2]. There are substantial economic links between these two zones, and in contemporary U.S. cities commuting occurs in both directions[24]. Northeast U.S. cities that developed before the automobile typically follow this narrative. Many have a relatively dense urban core, but have adopted zoning policies that ensure contemporary suburban settlements occur at lower density [23]. While they remain dense compared to other U.S. cities, they are getting less dense over time, as proportionally more of the population is in suburban areas. The declining manufacturing cities of the Rust Belt and the Southern Appalachians are an extreme example of this spreading out of population.

Southeastern U.S. cities, excluding Florida, are often newer and have less of a legacy of a dense urban core. They do not appear to be getting markedly denser, and the relatively fast population growth of these cities implies that their total impact on natural habitat in coming decades will be large. In contrast to the Southeast, Western cities appear to be getting denser, including those that do not have a historical legacy of a dense urban core such as Phoenix. These Western cities are often still growing quickly and consuming a great deal of land, but contemporary development is making these cities denser than they were previously. Many of these Western cities have a strong conservation culture, and the degree of conservation funding and reform-minded zoning correlates with how much denser they are getting. However, it should be noted that contemporary development in Western cities is still well below the densities found in the dense urban core of Northeastern U.S. cities, posing problems for designing effective public transit systems [25].

Interestingly, measurements of land inequality show that low-density neighborhoods that house a small proportion of Americans contain much of the developed area of our cities. This suggests that the preferences and economic choices of a relatively small number of urban residents are associated with much of the natural land-cover lost to development. Efforts to increase the density of existing neighborhoods (i.e., densification) may reduce urban expansion somewhat, but our results suggest that the strength of this effect will be diminished because a relatively small proportion of urban residents still desire to live in a more suburban setting and choose housing accordingly [26]. Public policy efforts to promote conservation may need to more directly consider the actions of this subset of residents causing most natural habitat loss in order to limit the impact of urban development on natural systems.

## Materials and Methods

### Data Sources and Preparation

For our analyses we defined urban areas using the 2003 metropolitan statistical areas (MSA) and consolidated metropolitan areas (CMSA) of the US Census Bureau [27]. Each MSA has a core area containing a substantial population plus additional adjacent areas that have an economic interaction with core, as defined by commuting data. Boundaries of MSAs generally follow county boundaries. Boundaries are selected so that the vast majority, although not all, of urban commuting occurs within an MSA. Throughout our analysis, we have used the 2003 MSAs so that the boundaries of an MSA do not change over time. More importantly, we use metrics in our analysis that are relatively insensitive to the location of the boundary of an urban area, to insure that any other definition of urban that includes the urban core plus surrounding commuting areas would yield quantitatively similar results to ours.

Land-cover data for our analysis was taken from the National Land Cover Database (NLCD) change product, which shows land-cover in 1990 and 2000 using a consistent classification methodology. NLCD information derived from a supervised classification of Thematic Mapper imagery (30m resolution) to a modified Anderson [28] Level 1 classification scheme (water, urban, barren/desert, forest, grassland/shrublands, agriculture, wetlands, ice/snow). Desert scrublands with some vegetative cover are placed in the grassland/shrubland category. In our analysis we primarily are interested in four types of land-cover transitions: agriculture to urban, forest to urban, wetland to urban, and grassland/shrublands to urban. All four of these land-cover transitions are specific types of urban expansion, as contrasted with densification, the process by which previously urban areas begin to house more people.

Our data on population and household density is from the Wildland-Urban Interface Database, version 2 [4]. This database contains US Census data from 1990 and 2000 at the census block level of aggregation, modified slightly to insure cross-census consistency of geographic units. As we have information on housing changes within many census blocks per MSA, this dataset allows analysis of process of densification as well as urban expansion. Census blocks are irregularly shaped and vary greatly in size, from less than 0.1 ha in dense urban areas to greater than 10 ha in rural areas with few people.

Information on all open space initiatives passed by municipalities is maintained by the Trust for Public Land (Landvote). We calculated the total conservation funding for each MSA from municipal and county ballot initiatives for the period 1990–2000. Note that we summed up total conservation funding in each MSA as the amount passed by each component county or municipality within the MSA. We then calculated average per-capita conservation funding for the MSA by dividing by its total population. This approach is appropriate for our comparative analysis of America's MSAs, but does average over variation in conservation funding within MSAs.

Information on general zoning patterns for the largest MSAs was taken from a large study by the Brookings Institution [23]. After a thorough review of all zoning laws in the thousands of constituent municipalities that make up these MSAs, Pendall et al. classified the 50 largest MSAs into 4 main categories (Figure 1): “Traditional”, where MSAs are made up of many constituent local municipalities, and planning and zoning standards have not been revised significantly since the 1920s; “Exclusion”, where municipalities commonly use measures to restrict apartment construction; “Wild Wild Texas”, where zoning is weak or non-existent; and “Reform”, where municipalities have moved beyond traditional zoning tools to other tools such as affordable housing measures, urban growth boundaries, or building-permit caps. In cases where metropolitan areas crossed state lines, Pendall et al. classified each sub-area separately. In our study, we have classified an entire MSA as one of the four categories, using the category of the majority of the urban area in the Pendall et al. study. For example, Pendall et al. classified most of the New York MSA as “Traditional,” but classified the New Jersey suburbs as “Exclusion.” In our study, the entire New York MSA is classified as “Traditional.” This reduces the number of MSAs in our study with a zoning category to 49.

### MSA-Wide Analysis

All data was clipped to MSA boundaries and projected to an Albers Equal-Area projection. For each MSA we tabulated open space loss and population change. The total open space lost, summing over all four categories, was correlated with the change in population as well as the median house value of the MSA, as determined by the U.S. Census. One simple measure of the tendency toward sprawl is simply the change in the per capita land consumption, in m^2^/person:
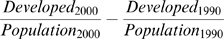



An increase in this number implies a city is getting more spread out, while a decrease means a city is getting denser.

### Fine-Scale Analysis

To gain a more detailed understanding of both urban expansion and densification, we classified census blocks into categories of household density (e.g., 0.5–1.0 housing units/km^2^). We converted the polygon Wildland-Urban Interface Database to a 30m raster representation, and calculated the total developed area and number of households in each household density category. From these data we could calculate the proportion of urban residents who live in dense neighborhoods (>1250 units/km^2^), as well as the change in this proportion over the decade.

Income inequality is often defined as the disparity in levels of income among individuals. An economy might have all workers earning similar incomes (low inequality) or have a few workers earning significantly more than others (high inequality). In an analogous fashion, land inequality may be quantified as the disparity in land consumption among households. A city might have all households consuming similar amounts of land (low inequality) or have a few households consuming significantly more than others. We quantify land inequality using the Gini index [29], which is commonly used to measure income inequality. The Gini index varies from 0 (all households “consume” developed area equally so that they are all in neighborhoods with identical developed area per capita) to 1 (a theoretical upper limit where one household consumed all developed land and the other households used no land).

### Statistical Analysis

Both MSA-wide metrics and fine-scale metrics were correlated with potentially explanatory continuous variables using the standard Pearson product-moment correlation coefficient. Zoning and the amount of public funding were treated as categorical variables (see the Results section for levels of each category), and the relationship between MSA-wide metrics and fine-scale metrics and these categorical variables was assessed with two separate one-way ANOVAs. If an ANOVA was statistically significant overall, differences among groups was evaluated using Tukey's Honestly Significant Difference (HSD) test. Only differences among groups significant at the P<0.05 level are reported in the paper. All statistical testing was done in the R software package.

## References

[1] Hobbs F, Stoops N (2002). Demographic Trends in the 20th Century..

[2] Jackson K (1985). Crabgrass Frontier..

[3] Theobald DM (2005). Landscape patterns of exurban growth in the USA from 1980 to 2020.. Ecology and Society.

[4] Radeloff VC, Hammer RB, Stewart S, Fried J, Holcomb S (2005). The wildland-urban interface in the United States.. Ecological Applications.

[5] Luck GW (2007). A review of the relationships between human population density and biodiversity.. Biological Reviews.

[6] McDonald RI, Kareiva P, Forman R (2008). The implications of urban growth for global protected areas and biodiversity conservation.. Biological Conservation.

[7] McDonald RI (2008). Global urbanization: Can ecologists identify a sustainable way forward?. Frontiers in Ecology and the Environment.

[8] Grimm NB, Faeth SH, Golubiewski NE, Redman CL, Wu JG (2008). Global change and the ecology of cities.. Science.

[9] McDonald RI, Urban DL (2006). Edge effects on species composition and exotic species abundance in the North Carolina Piedmont.. Biological Invasions.

[10] Hansen AJ, DeFries R (2007). Ecological mechanisms linking protected areas to surrounding lands.. Ecological Applications.

[11] Forman R (2008). Urban Regions: Ecology and Planning Beyond the City..

[12] Danielsen K, Lang R (1999). Retracting suburbia: Smart growth and the future of housing.. Housing Policy Debate.

[13] Porter D, Dunphy R, Salvesen D (2002). Making smart growth work..

[14] Calthorpe P (1993). The Next American Metropolis: Ecology, Community, and the American Dream..

[15] Duany A, Plater-Zyberk E, Speck J (2000). Suburban Nation: The Rise of Sprawl and the Decline of the American Dream..

[16] Talen E (2005). New Urbanism & American Planning: The Conflict of Cultures..

[17] Fulton W, Pendall R, Nguyen M, Harrisson A (2001). Who sprawls most? How growth patterns differ across the U.S..

[18] Theobald DM (2001). Land-use dynamics beyond the American urban fringes.. Geographical Review.

[19] Walker R (2001). Urban sprawl and natural areas encroachment: linking land cover change and economic development in the Florida Everglades.. Ecological Economics.

[20] Handy S (2005). Smart growth and the transportation - Land use connection: What does the research tell us?. International Regional Science Review.

[21] MacDonald K, Rudel T (2005). Sprawl and forest cover: what is the relationship?. Applied Geography.

[22] Irwin EG, Bockstael NE (2007). The evolution of urban sprawl: Evidence of spatial heterogeneity and increasing land fragmentation.. Proceedings of the National Academy of Sciences of the United States of America.

[23] Pendall R, Puentes R, Martin J (2006). From traditional to reformed: A review of the land use regulations in the nation's 50 largest metropolitan areas..

[24] Pisarski (2006). Commuting in America III: The Third National Report on Commuting Patterns and Trends..

[25] Kenworthy J, Laube F (1999). Patterns of automobile dependence in cities: an international overview of key physical and economic dimensions with some implications for urban policy.. Transportation Research Part A-Policy and Practice.

[26] Brown DG, Robinson DT (2006). Effects of heterogeneity in residential preferences on an agent-based model of urban sprawl.. Ecology and Society.

[27] Office of Management and Budget (2000). Standards for defining metropolitan and micropolitan statistical areas.. Federal Register.

[28] Anderson JR, Hardy EE, Roach JT, Witmer RE (1976). A land use and land cover classification system for use with remote sensor data..

[29] Gini C (1921). Measurement of Inequality of Incomes.. The Economic Journal.

